# Cutaneous Fusariosis in a Patient with Job's (Hyper-IgE) Syndrome

**DOI:** 10.1155/2020/3091806

**Published:** 2020-06-15

**Authors:** Ahmed M. Altibi, Radhika Sheth, Ayman Battisha, Vivek Kak

**Affiliations:** ^1^Henry Ford Allegiance Health (HFAH), Jackson, MI, USA; ^2^Harvard T. H. Chan School of Public Health, Harvard University, Boston, MA, USA; ^3^University of Massachusetts Medical School-Baystate, Springfield, MA, USA

## Abstract

*Fusarium* is a filamentous fungus that is ubiquitous in nature and can cause severe opportunistic infections in immunocompromised hosts. The association between *Fusarium* and hyper-IgE syndrome is exceedingly rare and has only been documented in a single report previously. A 44-year-old male, working as marijuana grower, with prior diagnosis of hyper-IgE syndrome and recurrent infections presented with enlarging right knee ulcer that did not respond to antimicrobial treatment. The patient was diagnosed with cutaneous fusariosis, confirmed with punch biopsy and positive wound cultures. The patient was managed with extended antifungal therapy (i.e., posaconazole) and surgical debridement resulting in remarkable improvement with wound healing leaving a pale scar. *Fusarium* should be considered in differential for cutaneous and invasive fungal infections in presence of cutaneous manifestations. Exposure to *Cannabis* plants is a noticeable risk factor. Multimodal approach involving systemic antifungals and wound debridement is essential for favorable outcome. Posaconazole was demonstrated to be a highly efficacious antifungal choice.

## 1. Introduction

*Fusarium *is a genus of filamentous fungi that can cause superficial, locally invasive, or disseminated infections in humans [1, 2]. Superficial infections typically occur in immunocompetent individuals, with the two classic infections being fungal keratitis and onychomycosis [3, 4]. On the contrary, invasive and disseminated infections occur almost exclusively in immunocompromised, particularly in profoundly neutropenic and T-cell deficient individuals. Patients with hematological malignancies and hematopoietic stem cell transplant recipients represent the majority of severe reported fusarial cases [5]. In immunocompromised individuals, the most common presentation for fusarial infections is prolonged fever refractory to antimicrobials in the setting of profound neutropenia; hence, it should be considered in differential for neutropenic fever. Other forms of infections include cutaneous fusariosis, sinusitis, pneumonia, endophthalmitis, and fusarial fungemia [6–8].

Cutaneous fusarial infections can be localized or disseminated. While the vast majority of immunocompromised individuals (88%) present with disseminated cutaneous lesions, only a minority of immunocompetent individuals (7%) develop disseminated cutaneous lesions [9]. Cutaneous involvement in the setting of an invasive fungal infection (e.g., fungemia) should raise suspicion for *Fusarium* as a potential culprit pathogen since skin involvement exists in 70% of patients with fusariosis [9]. The most commonly reported skin manifestations in cutaneous fusariosis include multiple painful erythematous papules/nodules, with or without central necrosis. Necrotic lesions tend to be associated with lesions resembling ecthyma gangrenosum, some with evolution into target lesions [9]. Other less common cutaneous manifestations include onychomycosis and periungual cellulitis.

In this clinical report, we describe a rare case of cutaneous fusarial infection in a patient with hyper-IgE syndrome (i.e., Job's syndrome). The association of fusarial infection with this rare immune disorder had only been previously reported in a single report in the literature [10]. We also shed light on successfully treating this case with oral posaconazole as monotherapy for fusariosis.

## 2. Case Report

**Figure 1:**
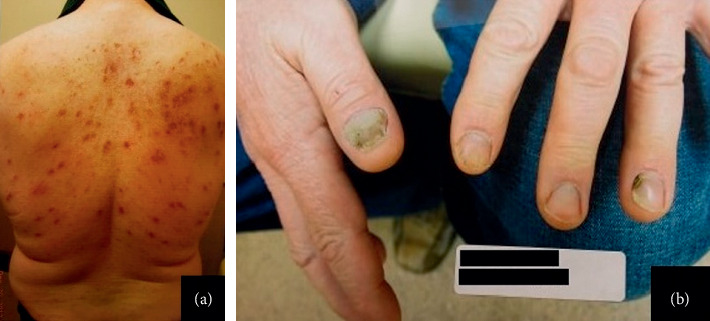
(a) Several scars on the patient's back resembling healing of old staphylococcal furunculosis; (b) distal subungual onychomycosis affecting multiple fingernails.

This is a 44-year-old Caucasian male patient with past history significant for hyper-IgE syndrome (confirmed with positive STAT3 mutation) and recurrent infections since childhood, including soft tissue infections (i.e., abscesses and osteomyelitis), recurrent pulmonary infections (i.e., empyemas), and recurrent staphylococcal infections. The patient presented to the infectious disease clinic with a progressively enlarging skin lesion on the lateral aspect of the right knee for two months. The lesion initially started as a quarter-sized ulcerative lesion associated with intermittent purulent discharge and pain upon palpation. Initial evaluation showed a temperature of 36.6°C (97.7°F), blood pressure of 108/60 mmHg, pulse of 80 beats/minute, and respiratory rate of 16/minute. On examination, the patient was well-developed and well-nourished. Cardiopulmonary and abdominal examinations were unremarkable. Upon skin inspection, the patient had several scars on his neck and back (Figure 1(a)), reflecting prior staphylococcal furunculosis previously treated with linezolid. Remarkably, the patient also had onychomycosis (Figure 1(b)), affecting several fingernails and toenails.

**Figure 2:**
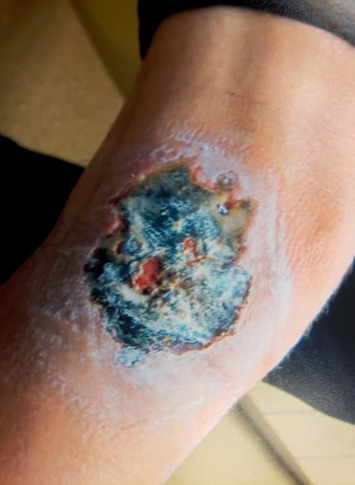
Ulcerative and necrotic lesion on the lateral aspect of the right knee measuring 7.5 × 6.0 × 0.1 cm. The wound base was mostly filled with eschar tissue with minimal granulation tissue.

The primary skin lesion on the lateral aspect of the right knee (Figure 2) was measuring 7.5 cm × 6.0 cm × 0.1 cm. The wound bed was mostly filled with eschar with very little granulation tissue. The surrounding skin was edematous, erythematous, and with mildly tender edges but did not show signs of cellulitis. Debridement was attempted during the initial encounter, but it was limited by the thick eschar tissue. Subsequently, wound culture and a punch biopsy of the lesion was obtained. The patient was scheduled for a follow-up visit with the wound clinic. Beyond the primary skin lesion, the patient denied having fever, chills, recent trauma, and animal/tick bites. He also denied a history of malignancy, chemotherapy, or hematopoietic stem cell transplant.

He has previously been treated for an HPV infection and had a negative hepatitis *C* and HIV screening tests recently. The patient had a recent travel to Hawaii one month prior to his initial encounter. During the trip, he indulged in aquatic sports, including swimming in pools and the ocean. Of notice, the patient has recently started a marijuana dispensary business, which also involved growing *Cannabis* plants. He reported that his marijuana plants were “damaged by mold” after which he had to bring new plants to grow.

### 2.1. Investigations

#### 2.1.1. Laboratory Findings

Initial workup showed a hemoglobin of 14.2 g/dl, white blood cell count of 3.9 k/ul (neutrophils: 56%, lymphocytes: 19%, monocytes: 15%, and eosinophils: 8%), and platelets of 214 k/ul. ESR and CRP were both within normal range at 13 mm/hour and <0.1 mg/L, respectively. Liver studies showed an ALT of 29 IU/L, AST of 35 IU/L, alkaline phosphatase of 57 IU/L, total bilirubin of 0.5 mg/dl, albumin of 5.0 g/dl, globulins of 3.4 g/dl, and total protein of 7.4 g/dl. Upon reviewing immunoglobulins levels, the patient was noticed to have a markedly elevated IgG (1,838 mg/dl, normally <200) and IgE levels (4,242 mg/dl, normally <300).

#### 2.1.2. Histopathology

**Figure 3:**
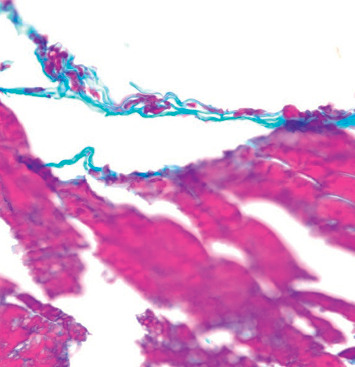
Punch excision skin biopsy obtained from a right lower extremity ulcer. Digestive PAS stain demonstrating the presence of numerous fungal hyphae consistent with fungal infection. Gram stain appeared negative for bacterial organisms.

A punch biopsy of the right knee lesion was performed during the initial encounter and showed an ulcer and abscess in the upper and mid-dermis. The deep dermis also exhibited suppurative inflammation within the subcutaneous tissue. Most remarkably, digestive PAS stain revealed the presence of numerous fungal hyphae consistent with deep fungal infection (Figure 3). Gram stain appeared negative for bacterial organisms.

#### 2.1.3. Microbiology

Wound cultures from the right knee lesion obtained on initial encounter isolated *Fusarium* species on the aerobic culture. However, no bacterial growth was observed on Gram stain or anaerobic cultures. Wound swab culture was repeated four days later which again identified *Fusarium* species as well as coagulase-negative staphylococci, likely representing cross contamination.

Sabouraud dextrose agar (SDA), the standard media for most fungi, was used to cultivate the organism. The VITEK-2 automated system was utilized for fungal identification, and *Fusarium* species were identified. In addition, lactophenol cotton blue (LPCB) wet-mount preparation was used for direct visualization of fungal structures, and structures consistent with *Fusarium* species were recognized (e.g., *Fusarium* species hyphae) under slide preparation. Further identification of the fusarial subspecies was attempted; however, the cost of processing was prohibitive.

### 2.2. Treatment and Outcomes

**Figure 4:**
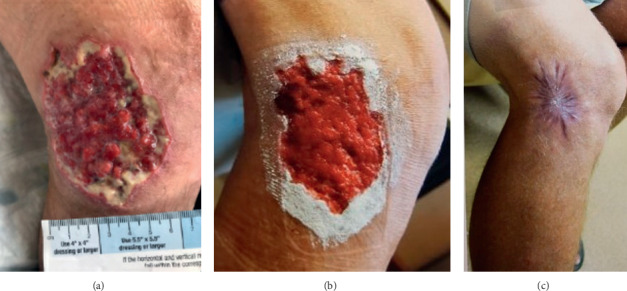
(a) Granulation tissue started to appear and serosanguineous discharge continued to show on the ulcer base (8.1 × 6.4 cm). (b) Progressive healing evidenced by hypergranulation with sloughing around the periphery (no serosanguinous discharge and no eschar tissue). (c) The size of the lesion had significantly reduced to 1.8 × 1.4 cm, and the wound healed completely leaving a pale and raised scar tissue at the site of the lesion.

Since *Fusarium* species were isolated from wound culture, the patient was initiated on antifungal therapy with *posaconazole* (300 mg once daily) for a planned treatment duration of 4–6 months. Since azole antifungals are notorious for hepatotoxicity, patient's liver functions were monitored monthly and were persistently normal. In addition, serum levels of posaconazole were monitored during the treatment duration to ensure achieving the target therapeutic range (>0.7 mcg/ml). Wound debridement was attempted; however, it was initially limited by the thick eschar tissue (Figure 4).

One week after initiating treatment, granulation tissues started to appear, and serosanguinous discharge continued to show on the ulcer base (Figure 4). On subsequent visits, surgical debridement was performed until almost all eschar tissue was removed. A month later, the ulcer showed hypergranulation with sloughing around the periphery, without eschar tissue or serosanguinous discharge (Figure 4). The patient continued to be free of symptoms during that time and was able to tolerate posaconazole without adverse events. After six weeks of treatment, the ulcer had reduced to 6.0 × 4.0 cm with visible pink granulation tissue at the base; no further surgical debridement was needed. By the third month, the wound healed completely leaving pale and raised scar tissue at the site of the lesion (Figure 4). The patient continued on *posaconazole* for a total of four months before it was discontinued.

## 3. Discussion

*Fusarium* is an opportunistic fungal pathogen that can cause localized, invasive, or disseminated infections in severely immunocompromised (IC) individuals [6]. The two most commonly reported risk factors for invasive fusariosis include neutropenia and hematological malignancies. In this article, we describe a case of cutaneous fusariosis in a patient with AD-HIES (a.k.a. Job's syndrome). While patients with Job's syndrome are prone to invasive opportunistic infections, an infection with fusariosis is an exceedingly rare event that had only been documented in a single report in the literature [10].

Individuals with hyper-IgE syndrome have a genetic mutation of the signal transducer and activator of transcription 3 (*STAT3*), a component of the *JAK-STAT* pathway that is required for the activation of a number of different immune cells [11]. A defect in *STAT3* impairs the cytokine signaling for differentiation of Th17 cells and chemotaxis of neutrophils [12]. Hence, patients with defective *STAT3* are susceptible to staphylococcal and fungal infections. Our patient had been suffering from recurrent staphylococcal abscesses, empyemas, and recurrent osteomyelitis since childhood—all attributed to his AD-HIES.

Interestingly, our patient has been growing marijuana in his home yard and reported that all his plants “died out because of the mold”. Hence, it is possible that exposure occurred upon contact with the infested *Cannabis* plants in his yard with subsequent inoculation of the *Fusarium* spores. The unique route of exposure to this rare fungus in the setting of an equally rare immunodeficiency disorder (i.e., Job's syndrome) is what makes this case intriguing.

While interrupting culture results, one should be thoughtful about the clinical context of these results [2]. For instance, *Fusarium* species growing in skin scraping of an immunocompetent patient likely represents contamination or colonization, whereas *Fusarium spp.* in respiratory secretion from an immunocompromised individual should be considered diagnostic for fusariosis [7]. *Fusarium* is also a well-known etiologic agent for onychomycosis responsible for 10–40% of cases caused by fungi other than dermatophytes [13, 14]. Fusarial infection in our case was associated with onychomycosis; however, a microbiologic confirmation for the causative organism was not sought.

*Fusarium* can invade blood vessels causing hematogenous spread—the hallmark of “disseminated” fusariosis [15]. A definitive diagnosis of “disseminated” disease requires two positive culture from either internal organs, blood cultures, or both [16]. On the contrary, a fusarial infection is classified as “local” when there is a histological evidence of invasion confirmed by a positive culture from the same site without systemic involvement [1]. In our case, the patient had histological and microbiological diagnosis for *Fusarium*, but with negative blood cultures or distant organ involvement; hence, the infection was classified to be “local”.

In histopathology sections, *Fusarium* species often appears similar to *Aspergillus* and *Pseudallescheria* species, with septate hyphae branching at an acute angle. It is important to distinguish between them as the latter two tends to be more resistant to the commonly used antifungals.

So far, there are no well-established treatment guidelines for fusarial infections, given the rarity of the disease and the species-dependent susceptibility to antifungals. Most data in the literature are derived from case reports and limited observational studies in immunocompromised patients [7]. In general, a successful treatment strategy entails a composite of systemic antifungals, surgical debridement, and source control (e.g., removal of potential source catheters). Fusarial species tend to be resistant to most antifungals in vitro; hence, the choice of antifungal can be challenging. While universally resistant to echinocandins (i.e., caspofungin), successful treatment with favorable outcomes had been reported with amphotericin B [12, 13, 17], voriconazole [18, 19], and posaconazole [20–22]. Optimum treatment duration remains obscure, but generally antifungals are recommended to be continued at least until all clinical signs of infection resolve. Patients with nonlocalized fusariosis typically require prolonged course of treatment (i.e., months). For instance, the case by Belcher et al. required eight months of aggressive systemic therapy, while in our case, treatment with oral posaconazole extended for a total of four months. Nonetheless, this can largely vary based on the extent of dissemination of infection and the immune status of the patient. Further studies are required to explore the ideal antifungal treatment and also the therapeutic efficacy of posaconazole in fusarial infections.

## 4. Conclusion

We highlighted a case of cutaneous fusariosis in the setting of a rare entity “Job's syndrome.” *Fusarium* species should especially be considered in the differential for local and invasive fungal infections in presence of cutaneous manifestations. In addition to immune deficiency, exposure to *Cannabis* plants might be a noticeable risk factor. A multimodal approach involving systemic antifungals and wound debridement is essential for a more favorable outcome. Fusarial infections tend to require prolonged treatment duration (i.e., months). Posaconazole was demonstrated in our case to be a highly efficacious antifungal choice. Yet, further large-scale studies are required to characterize the optimum antifungal regimen and duration of therapy.

## Data Availability

No data were used to support this study.
